# Endovascular Extraction of an Aortic Arch Thrombus Using Dual Snares and Aspiration Thrombectomy

**DOI:** 10.1016/j.jaccas.2025.105748

**Published:** 2025-10-25

**Authors:** Michal Droppa, Hüseyin Kocaman-Graf, Katharina Feil, Annerose Mengel, Ulrike Schempf, Ulf Ziemann, Reimer Riessen, Robert Rottscholl, Meinrad Gawaz, Tobias Geisler

**Affiliations:** a Department of Cardiology and Angiology, University Hospital Tübingen, Eberhard Karls University Tübingen, Tübingen, Germany; b Department of Neurology & Stroke, University Hospital Tübingen, Eberhard Karls University Tübingen, Tübingen, Germany; c Department of Internal Medicine I, University Hospital Tübingen, Eberhard Karls University Tübingen, Tübingen, Germany; d Department for Internal Medicine, Intensive Care Unit, University Hospital Tübingen, Eberhard Karls University Tübingen, Tübingen, Germany; e Department of Pathology, University Hospital Tübingen, Eberhard Karls University Tübingen, Tübingen, Germany

**Keywords:** aortic arch thrombus, aspiration thrombectomy, endovascular extraction

## Abstract

**Background:**

Aortic mural thrombi are rare causes of embolic stroke and pose therapeutic challenges, particularly in critically ill patients.

**Case Summary:**

A 47-year-old man presented with middle cerebral artery infarction. Imaging revealed a large, mobile mass in the aortic arch, suspected as the embolic source. Surgical removal was contraindicated owing to cerebral hemorrhage, and an endovascular approach was chosen. Using a cerebral protection device, dual snares, and a large-bore aspiration catheter, the thrombus was successfully removed without procedural complications. Histology confirmed an organized thrombus. Despite technical success, the patient died from complications of malignant stroke.

**Discussion:**

This case highlights a unique endovascular strategy for removing an intra-aortic structure. The combined use of aspiration and snaring devices enables safe extraction, with cerebral protection minimizing embolic risk. This approach may serve as a valuable alternative to surgery in selected high-risk patients.

**Take-Home Message:**

Minimally invasive removal of aortic thrombi is feasible and may reduce embolic risk in critically ill patients.

## Graphical Abstract

**Figure:**
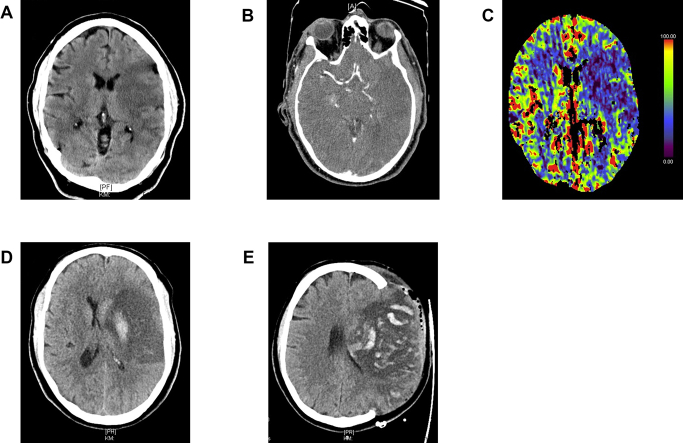

## History of Presentation

**Figure 1:**
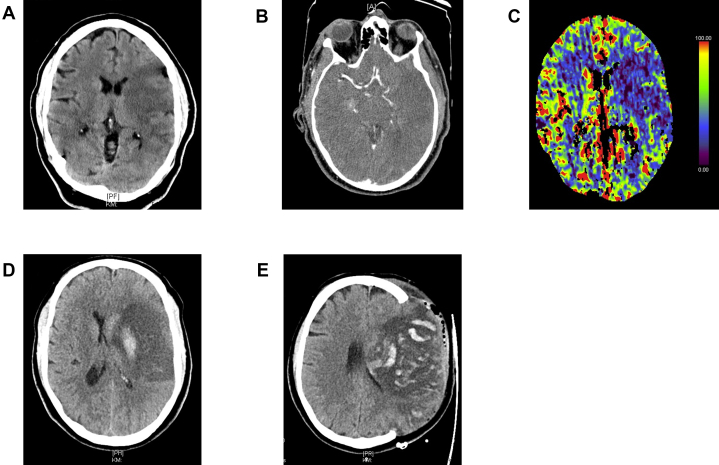
Computed Tomography Showing a Large Infarction of the Left Middle Cerebral Artery (A) Baseline with early infarct demarcation. (B) Angiography showing occlusion in the M1 segment. (C) Perfusion mismatch. (D) Follow-up 1 day later showing progressive cerebral edema. (E) After decompressive hemicraniotomy with secondary bleeding in the infarction.

A 47-year-old man without any relevant prior medical history or functional limitations (prestroke modified Rankin scale: 0 points) was referred to our neurological clinic. He presented with right-sided hemiparesis, neglect, and global aphasia (National Institutes of Health Stroke Scale: 21 points, Glasgow Coma Scale: 13 points) with unknown symptom onset (last seen well the day before). Initial cranial computed tomography (CT) with CT angiography and perfusion revealed early infarct demarcation in the left middle cerebral artery (MCA) territory (Alberta Stroke Program Early CT Score [ASPECTS]: 6), large vessel occlusion in the M1-segment of the MCA, and perfusion mismatch (Figures 1A to 1C). Therefore, intravenous thrombolysis was not possible, but endovascular thrombectomy was performed, and the patient was admitted to the neurological intensive care unit.

**Take-Home Messages**

• This case highlights the successful interventional removal of an aortic thrombus using a cerebral protection device, dual snares, and a large aspiration catheter.
• Interventional removal of an intra-aortic mass may represent a promising alternative for patients with a high risk of thromboembolic events and elevated surgical risk.

Despite sufficient reperfusion (modified treatment in cerebral infarction [mTICI] score: 2a), the patient's condition deteriorated with anisocoria and decreased consciousness, and he developed a malignant MCA infarction (Figure 1D), necessitating decompressive hemicraniotomy the following day. Postoperatively, the patient's course was complicated by recurrent intracranial pressure (ICP) crisis, requiring intensive neurocritical care. Follow-up imaging revealed secondary intracerebral hemorrhage in the infarcted area (Figure 1E), which was managed conservatively.

## Investigations and Differential Diagnosis

**Figure 2:**
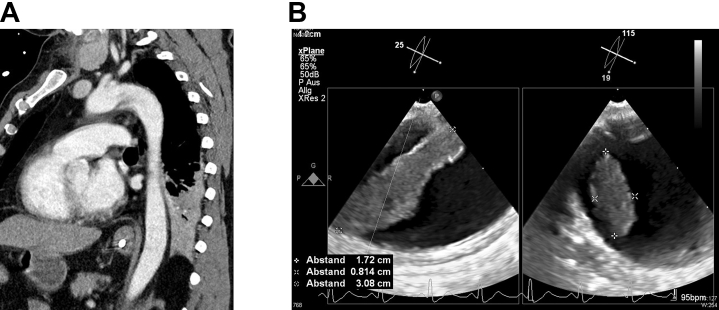
Intravascular Mass in the Aortic Arch (A) Computed tomography scan. (B) Transesophageal echocardiography.

Further investigations, including CT angiography of the aorta, identified a large structure in the aortic arch, suspected as a possible source of embolic stroke. A thrombus or primary malignancy was considered. Whole-body CT scanning found no malignancy in other organs, although a small embolic infarction in the spleen was detected. Transesophageal echocardiography showed a mobile structure measuring approximately 3 × 2 × 0.8 cm originating from the aortic wall in the aortic arch (Figure 2). Given the high mobility of this structure and the associated risk of further embolization, as well as the need for histological analysis, removal was indicated. However, because of the patient's critical condition and contraindications for extracorporeal circulation stemming from the cerebral bleed, surgical removal was ruled out. Thus, an interventional approach to remove the structure was planned. The decision to proceed with interventional removal was made at a time when the neurological prognosis was still uncertain and recovery potential remained.

## Management

Initially, a cerebral protection device (Sentinel, Boston Scientific) was deployed through the right radial artery. Both femoral arteries were punctured, and a 24-F sheath was placed on the right side, with a 16-F sheath on the left. An endoscopic snare was deployed in the descending aorta and opened. A 24-F aspiration thrombectomy system (FlowTriever, Inari Medical) was then advanced into the descending aorta through the endoscopic snare, with a secondary snare inserted through the aspiration system. The aspiration catheter was positioned adjacent to the structure, and under transesophageal echocardiography guidance, the structure was snared through the aspiration catheter. The aspiration catheter was then advanced over the snare toward the structure, and aspiration was applied.

**Figure 3:**
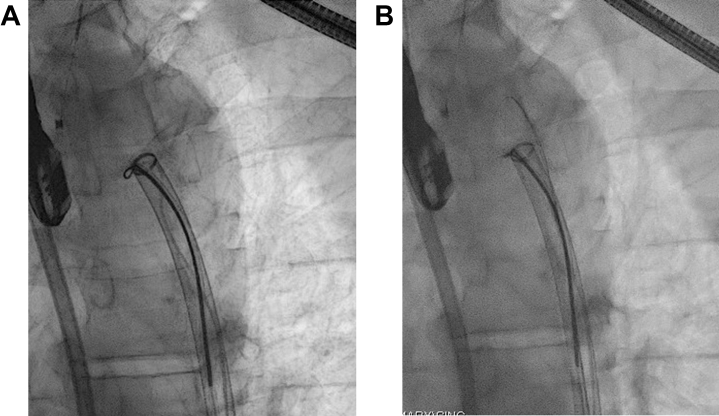
Extraction of the Intravascular Mass (A) Snaring of the mass through the aspiration catheter. (B) Detachment of the structure from the aortic wall using the endoscopic snare.

**Figure 4:**
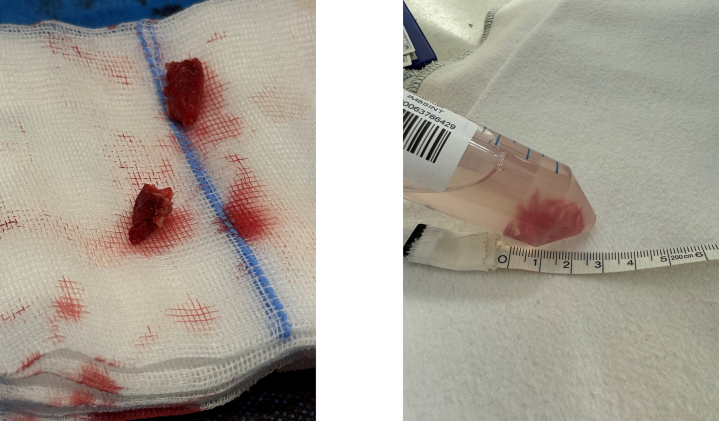
Tumor Retrieved From the Aspiration Catheter

**Figure 5:**
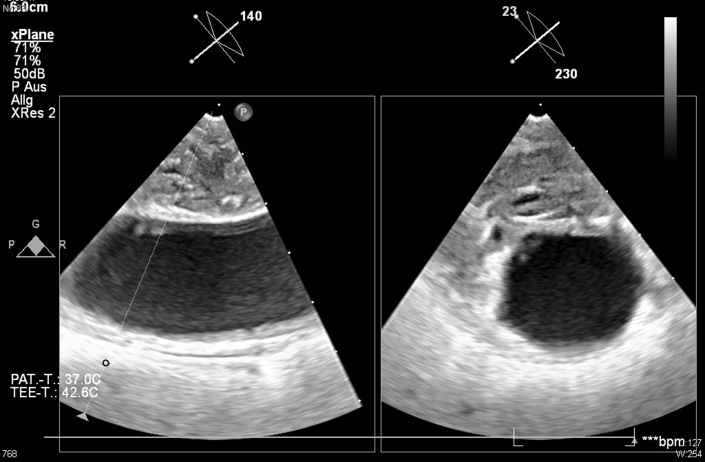
Transesophageal Echocardiography After Mass Removal

When the structure did not detach, the previously placed endoscopic snare was advanced over the aspiration catheter to the aortic wall, where the structure was anchored. The endoscopic snare was then closed, detaching the structure from the aortic wall. Although it was possible to apply electricity for an electrosurgical cut, this was unnecessary, as the structure released easily upon closure of the snare. Subsequently, the structure could be fully aspirated into the catheter (Figure 3, Video 1). Three fragments of the solid tumor were retrieved from the catheter for histological analysis (Figure 4). Echocardiography revealed only minimal remains of the structure on the aortic wall (Figure 5). Both femoral arteries were closed with a percutaneous suture-mediated closure system (2 Abbott ProGlide devices per side). The cerebral protection device was removed, with no embolic material detected. No complications occurred during or after the procedure.

## Outcome and Follow-Up

**Figure 6:**
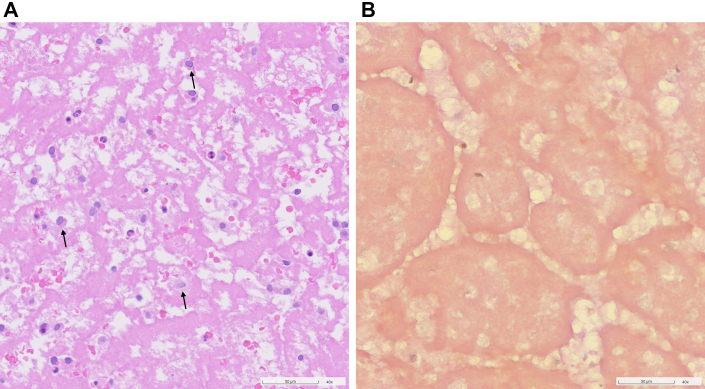
Histologic Images of the Resection Specimen (A) Hematoxylin and eosin stain showing old fibrin with scattered macrophages (arrows) and erythrocytes. Significant inflammatory infiltrates were absent. (B) Elastica van Gieson stain: no collagenous fibers could be detected, thus no signs of organization were noted.

Histologic work-up of the specimen revealed an older thrombus with first signs of resorption, but features of more advanced organization of the mass were absent. No significant inflammatory infiltrate was detected (Figure 6).

The postprocedure course was complicated by acute kidney failure, requiring dialysis, as well as liver failure. Six days after thrombus removal, the patient experienced a sudden increase in ICP caused by subgaleal bleeding. During emergency surgery, diffuse bleeding was observed. Subdural and epidural hematomas were evacuated; however, adequate hemostasis could not be achieved, and ICP remained critically elevated. Given the poor prognosis, therapy was de-escalated, and the patient died 9 days after thrombus removal.

## Discussion

We present a challenging case of the interventional removal of a large intravascular mass from the aortic arch. Using a cerebral protection system, an aspiration thrombectomy catheter, and dual snares, we successfully extracted the structure without complication.

Several options exist for the interventional removal of intravascular masses. Dedicated thrombectomy devices, such as the AngioVac^1^, ^2^, ^3^ or Penumbra Lightning System,^4^ have been proven effective in removing intracardiac and intravascular masses. The ŌNŌCOR device has been used to remove an intracardiac mass with the assistance of a snare,^5^ and more recently, a combination of the ŌNŌCOR device and an electrocautery endoscopic snare was successfully used to remove a right atrial tumor.^6^

In this case report, we describe a minimally invasive approach using the FlowTriever system in combination with 2 snares for the removal of a mass in the aortic arch, similar to the approach described by Steinberg et al.^6^ The FlowTriever aspiration device is primarily designed for treating pulmonary emboli.^7^ It features a large 24-F flexible catheter for aspiration, with the capability of autologous blood return. Unlike other devices, it avoids the use of an extracorporeal circuit with a centrifugal pump, thereby minimizing rapid blood loss and simplifying handling.

Previously, we reported the removal of a left ventricular mass using a large steerable sheath and snare.^8^ Incorporating a second endoscopic snare during mass removal enables more precise detachment of the structure and facilitates electrocautery-based removal. This approach allows for complete removal of the mass, potentially reducing the risk of embolization associated with partial debulking. In our opinion, this dual-snare approach should be preferred in cases with firm attachment or brad-based lesions, whereas a simpler configuration with a single snare may be sufficient in other anatomical situations. However, in cases involving the aorta or left-sided heart structures, the use of a cerebral protection device, as demonstrated in our case, is strongly recommended. We used the Sentinel device via right radial artery access; however, full cerebral protection covering the left subclavian and consequently the vertebral artery would have been the preferable option, but such a device was not available at our institution.

Aortic mural thrombus is a rare cause of systemic embolism, typically occurring in the aortic arch and associated with potentially fatal complications and significant mortality.^9^^,^^10^ Atherosclerosis, thrombophilia, or other hypercoagulable conditions, such as malignancy or rheumatologic disorders, may contribute to the pathogenesis of aortic thrombi; however, the exact etiology often remains unclear. The exact etiology of the large thrombus in our patient remains unclear. A comprehensive malignancy screening, including whole-body CT, was negative. The patient had no history of trauma, infection, or prior surgery, and there were no signs of systemic inflammatory or autoimmune disease. Although hypercoagulability due to occult disease cannot be fully excluded, the thrombus might have been triggered by local endothelial injury, small noncalcified atherosclerotic plaque, or a transient prothrombotic state.

## Conclusions

Conservative therapy with anticoagulation may be the preferred treatment option for patients with aortic thrombi without a high risk of embolization. In contrast, for patients at high risk of recurrent embolic events, such as those with large, mobile thrombi, aortic atherosclerosis, or a history of stroke, a more aggressive approach such as interventional or surgical thrombus removal may be warranted. However, surgical treatment requires extracorporeal circulation and deep hypothermic circulatory arrest, which pose significant perioperative risks, especially for critically ill patients or those with acute stroke.

## Funding Support and Author Disclosures

Support was received by the Open Access Publishing Fund of the University of Tübingen. The authors have reported that they have no relationships relevant to the contents of this paper to disclose.

## Appendix

Video 1 Transesophageal Echocardiography Showing Aspiration of the Structure Into the Aspiration Catheter
